# Dissemination of Class A Cephalosporinases and Class D Carbapenemases in Escherichia coli Isolates From a Tertiary Hospital in Sudan

**DOI:** 10.7759/cureus.44365

**Published:** 2023-08-30

**Authors:** Khalid E Khalid

**Affiliations:** 1 Department of Basic Medical Sciences, Faculty of Applied Medical Sciences, Al-Baha University, Al-Baha, SAU

**Keywords:** multiplex pcr, urinary tract infections, esbl, multi-drug resistance, carbapenemases, cephalosporinases, escherichia coli

## Abstract

Introduction

The high prevalence of urinary tract infections (UTIs) and rising resistance to beta-lactam antibiotics, which is a global therapeutic concern, are caused by *Escherichia coli* (*E. coli*) extended-spectrum beta-lactamases (ESBLs) producers. It is unclear how *E. coli* that produces ESBLs spreads throughout Gezira state, Sudan. The study aimed to evaluate the dissemination of class A and class D resistance genes among *E. coli* and to recognize the antibacterial activity of the locally used cephalosporins and carbapenems.

Methods

One hundred and fifteen isolates of uropathogenic *E. coli* were collected from patients who attended a tertiary hospital. The isolates were identified using colony morphology, gram staining, and biochemical tests and checked for 16S rRNA using PCR. The multidrug-resistant (MDR) testing was conducted using agar disk diffusion. Finally, the class A and D resistance genes were analyzed by multiplex PCR.

Results

The study enrolled 200 patients with UTIs. *E. coli* isolates were found in 115 (57.5%) urine specimens examined, and 60 (52.2%) of them produced resistance to most locally used antibiotics. The antibiotic resistance pattern was higher against cefepime (100%), ceftizoxime (90%), cefuroxime (81.7%), and ceftriaxone (81.7%) and had lower activity against meropenem (13.3%). The genotypic characterization of class A cephalosporinases was 85% for *bla*_CTX-M_, 70% for *bla*_SHV_, and 33.3% for *bla*_TEM_, while for class D carbapenemases, it was 10% for both *bla*_OXA-23 _and *bla*_OXA-51_.

Conclusion

The considerable antibiotic resistance to the cephalosporins and meropenem and the increased predominance of the *bla*_CTX-M_ and *bla*_SHV _genes are serious concerns for the health authorities. Meropenem could still be used as the drug of choice for ESBL-producing *E. coli*.

## Introduction

*Escherichia coli* (*E. coli*) is a gram-negative opportunist pathogen in the human intestinal tract. When this bacterium gets into unnatural places, it can cause opportunistic infections and infections of the bloodstream, skin, soft tissue, sepsis, and urinary tract [1]. *E. coli*, which also has a high mortality and morbidity rate as well as significant economic expenditures related to its treatment, is the primary cause of about 85% of community-acquired and 50% of hospital-acquired urinary tract infections (UTIs) [1-3].

The antimicrobial resistance phenomenon has existed for a long time; however, the global dissemination of resistance has posed a challenge [4]. Enzyme-mediated resistance is a worldwide public health problem due to its rapid expansion and the generation of multidrug-resistant (MDR) bacteria that are increasingly difficult to eliminate.

Beta-lactamase enzymes were first described in 1940 in England, isolated from *E. coli*, which prompted antibiotic resistance research [5].

A group of these beta-lactamase enzymes is responsible for the current spread and increase of penicillin, carbapenem, and cephalosporin resistance. *E. coli* is essentially sensitive to almost all clinically related antibiotics, but this bacterium can accumulate antibiotic-resistant genes, mainly through horizontal gene transfer [6]. By making more and more extended-spectrum beta-lactamases (ESBLs), *E. coli* becomes more and more resistant to expanded-spectrum cephalosporins.

In the early 1980s, TEM and SHV beta-lactamases were found capable of hydrolyzing the beta-lactam ring of cephalosporins, and therefore, resistance was soon reported. Single-point mutations in these enzymes allowed beta-lactamases to break penicillin as well as the first-, second-, and third-generation cephalosporins, and even monobactams. By 1988 and 1989, the first isolate of SHV-ESBL was found in clinical samples from Argentina and Chile, respectively [5]. Since then, different types of enzymes have been detected, with CTX-M being the most common [7].

Carbapenems have attracted wide attention due to their high antimicrobial activity and low toxicity. However, *E. coli* has developed resistance to this drug, resulting in a major global health concern [1]. Even though many beta-lactamase-producing strains are still susceptible to carbapenems, the increased use of these drugs has led to a rise in the spread of enzymes that break down carbapenems. These enzymes are called carbapenemases [3,4], and they are found in many clinically important gram-negative species but not in *E. coli*. OXA enzymes, which belong to the class D lactamase, are invariably linked to modest degrees of resistance to carbapenems such as meropenem, imipenem, and doripenem [2,3,8]. For serious infections caused by *Enterobacteriaceae* that produce ESBL, carbapenems are still the medicine of choice among all beta-lactam antibiotics [2,9].

A previous study among our population found that *E. coli* is the most frequent gram-negative isolate (54%), and it is resistant to first-line antibiotics [10]. Other two studies [11,12] have been conducted in the Khartoum state of Sudan, where they found that the occurrence of ESBL-producing *E. coli* was 31% and 38%, respectively, with the predominance of the blaTEM gene (61% and 86%, respectively).

The detection of class A cephalosporinases and class D carbapenemases in ESBL-producing *E. coli* and their pattern of antibiotic resistance can tell us a lot about their epidemiology and help us come up with a better way to treat them. Drug resistance is becoming more common, making it take longer to treat patients, especially those in hospitals. Therefore, the purpose of this study was to assess the prevalence of *E. coli* strains that produce ESBL as well as the antibacterial activity of these strains against local cephalosporin and carbapenem antibiotics in UTI patients who were admitted to a tertiary hospital in Gezira state, Sudan.

## Materials and methods

Patients and sampling

This is a cross-sectional, descriptive, laboratory-based study. Two hundred urine samples were collected in the period between August 2022 and December 2022 from patients of all ages with symptoms of UTIs who attended the Wad Medani Teaching Hospital outpatient clinic in Gezira state, Sudan. This hospital is the largest governmental hospital outside the capital Khartoum, Sudan; it has around 254 beds and an average of 820 admissions per month. It provides free medical services for the people in Gezira state and subordinate cities, and it is considered an important tertiary hospital for the training of medical students, particularly those from Gezira University and other private medical colleges in the state.

The samples were collected in sterile, dry, wide-necked, and leakproof containers, and the patients requested 10-20 mL of specimens from the midstream urine. Urine samples were immediately sent for microbiological, biochemical, and molecular tests to the Department of Clinical Microbiology at the Faculty of Medical Laboratory Sciences (FMLS), University of Gezira (U of G), Wad Madani, Sudan. The samples tested positive for *E. coli* culture due to the presence of ³10^5^ colony-forming units (CFU) of *E. coli* bacteria per milliliter for midstream urine samples.

The patients were notified of the study’s purposes and objectives; consequently, informed consent was obtained in written form. Ethical permission was obtained from the ethical committee of the FMLS at the University of Gezira, Wad Madani, Sudan.

Bacterial isolation, gram staining, and biochemical tests

To find the UPEC, urine samples were cultured on MacConkey agar (Neogen, USA) and cystine lactose electrolyte deficient (CLED) agar (Sigma-Aldrich, USA). Culture plates were incubated for 24 hours at 37°C, and then, the growth colonies were identified using colony morphology, gram staining, and biochemical tests.

Dried smear slides were prepared from yellow medium colonies (lactose fermenters) for the gram staining procedure [13]. In brief, the dried smears were covered with crystal violet for one minute, followed by Lugol’s iodine solution for one minute, washed off with acid alcohol, stained with carbol-fuchsin stain solution for two minutes, and lastly, examined microscopically using a 100× lens.

Traditional biochemical tests, like the oxidase test, the indole test, urea broth media, Kliglar iron agar for citrate, and Simon citrate agar, were done on the pure isolates.

Antimicrobial susceptibility test

All of the UPEC isolates were tested to see if they were resistant to locally used antibiotics like meropenem (10 mg), ceftriaxone (30 mg), cefuroxime (30 mg), ceftizoxime (30 mg), and cefepime (30 mg) using the Kirby-Bauer disk diffusion method, as recommended by the Clinical and Laboratory Standards Institute [14]. The diameters of the inhibition zone were measured, and the isolates were categorized as susceptible, intermediate, and resistant.

Detection of phenotypic ESBLs

The screening of the phonotypic ESBL-producing isolates was conducted according to CLSI instructions. Disks containing cefepime (30 mg), ceftazidime (30 μg), and cefotaxime (30 μg) with or without clavulanic acid (10 mg) were used to examine the ESBL-positive isolates. The isolates were considered ESBL-positive. If the inhibition zone diameter around the combination disk is ≥5 mm, than the inhibition zone around the single disk of the same antibiotic, the* E. coli* standard strain ATCC 25922, was used as a negative control.

Characterization of ESBL-producing bacteria

Genomic DNA was extracted from bacterial colonies using the boiling lysis method [15]. The extracted DNA quantity and quality were determined using a NanoDrop spectrophotometer (Bibby Scientific, UK). Multiplex PCR was used to look for the *bla*_CTX-M_, *bla*_SHV_, *bla*_TEM_, *bla*_OXA-23_, and *bla*_OXA-51_ genes in ESBL-positive isolates.

As an internal control, 16S rRNA from bacteria was found using a single PCR reaction, and resistance genes were found using two different types of PCR reactions. The primer sequences designed for the detection of the resistant genes are presented in Table 1 (Macrogen, Seoul, South Korea) [16].

**Table 1:** Primer sequences and product sizes of 16S rRNA, blaTEM, blaSHV, blaCTX-M, blaOXA-23, and blaOXA-51 genes

Studied genes	Primer sequence	Product size	Reference
*bla*_CTX-M _-F *bla*_CTX-M _-R	5-CGACAGCTGGGAGACGAAAC-3 5-CGGTGGTATTGCCTTTCATCC-3	193 bp	This study
*bla*_SHV _-F *bla*_SHV _-R	5-AGGATGTATTGTGGTTATGCGTT-3 5-CGAGTAGTCCACCAGATCCT-3	332 bp	This study
*bla*_TEM_ -F *bla*_TEM_ -R	5-TGCTATGTGGTGCGGTATTATC-3 5-AACTTTATCCGCCTCCATCCA-3	425 bp	This study
*bla*_OXA-23 _-F *bla*_OXA-23 _-R	5-GAT CGG ATT GGA GAA CCA GA-3 5-ATT TCT GAC CGC ATT TCC AT-3	501 bp	Ref. [16]
*bla*_OXA-51 _-F *bla*_OXA-51 _-R	5-TAA TGC TTT GAT CGG CCT TG-3 5-TGG ATT GCA CTT CAT CTT GG-3	353 bp	Ref. [16]
16S rRNA -F 16S rRNA -R	5-AGGCCTTCGGGTTGTAAAGT-3 5-ACCTCCAAGTCGACATCGTT-3	420 bp	Ref. [16]

For the 16S rRNA, the PCR condition was started by initial denaturation at 95°C for six minutes, followed by 40 cycles of denaturation at 95°C for 30 seconds, annealing at 50°C for 50 seconds, extension at 72°C for 60 seconds, and final extension for 10 minutes at 72°C.

The multiplex PCR was optimized as follows: initial denaturation at 95°C for three minutes, 40 cycles of annealing at 95°C for 30 seconds, 52°C for 45 seconds, and 72°C for 45 seconds, followed by a final extension at 72°C for three minutes. The PCR products were separated on a 2% agarose gel and visualized under the UV gel documentation system (BioRad, USA).

Statistical analysis

The data were analyzed using IBM SPSS Statistics, version 23.0 (IBM Corp., Armonk, NY, USA). Categorical variables were presented as counts and percentages.

## Results

The 115 (57.5%) *E. coli* strains were isolated from 200 (113 females and 87 males) patients suffering from UTIs. Of the 115 *E. coli* isolates, 60 (52.2%) were MDR to cephalosporines and meropenem. The isolate was more prevalent in women (33 cases) than men (27 cases).

Ceftriaxone, cefuroxime, ceftizoxime, and cefepime were used to figure out the antibiogram profile of the *E. coli* isolates. Meropenem was used to represent the carbapenems. The antibiotic susceptibility pattern of the *E. coli* isolates showed the highest sensitivity toward meropenem (86.7%), followed by ceftriaxone, cefuroxime, and ceftizoxime (Table 2).

**Table 2:** Antimicrobial susceptibility patterns of Escherichia coli isolates to the common locally used antibiotics (N = 60)

Antibiotics	Antimicrobial susceptibility of *E. coli* isolates		
	Sensitive no (%)	Intermediate no (%)	Resistant no (%)
Meropenem (10 mg)	52 (86.7)	0	8 (13.3)
Ceftriaxone (30 mg)	9 (15.0)	2 (3.3)	49 (81.7)
Cefuroxime (30 mg)	5 (8.3)	6 (10)	49 (81.7)
Ceftizoxime (30 mg)	3 (5.0)	1 (1.7)	54 (90.0)
Cefepime (30 mg)	0	0	60 (100.0)

As shown in Table 3, the resistance profile of *E. coli* was higher against ceftriaxone (81.7%), cefuroxime (81.7%), ceftizoxime (90.0%), and cefepime (100.0%) and lower against meropenem (13.3%).

**Table 3:** The antibiotic resistance patterns of the Escherichia coli isolates according to the common locally used antibiotics (N = 60) F, frequency

Meropenem		Ceftriaxone		Cefuroxime		Ceftizoxime		Cefepime	
F	%	F	%	F	%	F	%	F	%
8	13.3	49	81.7	49	81.7	54	90.0	60	100

In the 60 ESPL-positive isolates, there were 51 (85.0%) phonotypic genes of class A cephalosporinases for *bla*_CTX-M_, 42 (70.0%) for *bla*_SHV_, and 20 (33.3%) for *bla*_TEM_. On the other hand, the number of phenotypic genes of class D carbapenemases was six (10%) for *bla*_OXA-23_ and six (10%) for *bla*_OXA-51_ (Table 4).

**Table 4:** The frequency (F) and percentage (%) of the genotypic-resistant genes among the ESBL-producing Escherichia coli isolates (N = 60)

Class A						Class D			
*bla*_CTX-M_		*bla*_SHV_		*bla*_TEM_		*bla*_OXA-23_		*bla*_OXA-51_	
F	%	F	%	F	%	F	%	F	%
51	85.0	42	70.0	20	33.3	6	10.0	6	10.0

**Figure 1:**
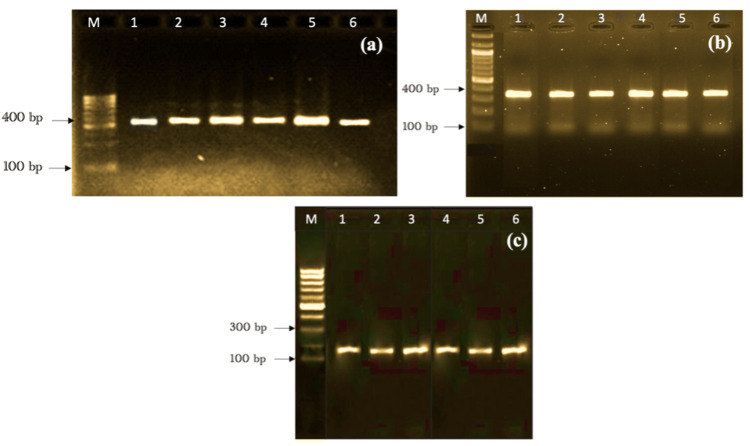
Detection of (a) blaTEM, (b) blaSHV, and (c) blaCTX-M genes in ESBL-producing Escherichia coli Lane 1: Markers (DNA ladder 100bp) Lanes 1-6: The PCR products of blaTEM (425 bp), blaSHV (332 bp), and blaCTX-M (193 bp) ESBL, extended-spectrum beta-lactamase

**Figure 2:**
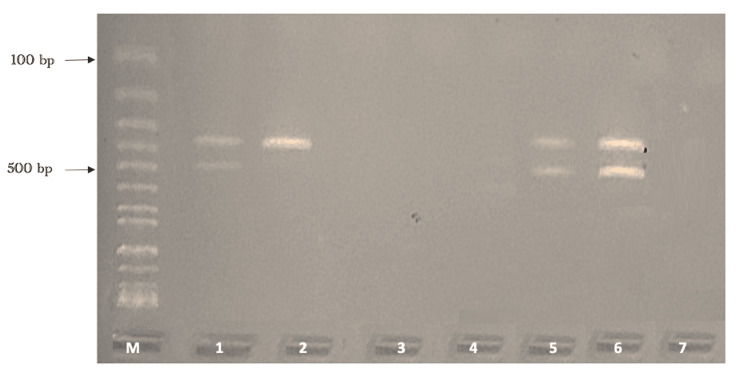
Detection of blaOXA-51 and blaOXA-32 genes in ESBL-producing Escherichia coli Lane 1: Marker (DNA ladder 100bp) Lanes 1, 6, 11, and 12: The PCR product of blaOXA-51 (353 bp) and blaOXA-23 (501 bp) ESBL, extended-spectrum beta-lactamase

Figure 1 shows how *bla*_CTX-M_ (193 bp), *bla*_SHV_ (332 bp), and *bla*_TEM_ (425 bp) are different at the molecular level. Figure 2 does the same for *bla*_OXA-51_ (353 bp) and *bla*_OXA-23_ (501 bp).

## Discussion

The prevalence of uropathogenic *E. coli* (UPEC) isolates has been extensively studied among different populations in different countries since this pathogen is the major cause of UTIs, accounting for about 85% of community-acquired and 50% of hospital-acquired infections, resulting in high rates of mortality and morbidity [1-4]. This made us want to find out how common it was in our population because we thought there would be a high rate of resistance to clinically related antibiotics used as a routine in our country. There were two reasons for this, and the first was that *E. coli* is said to be resistant to almost all clinically related antibiotics because it can store antibiotic resistance genes [6], and the second was the indiscriminate use of antibiotics for UTI treatment as a result of the permitted over-the-counter medicine (OCT) in Sudan.

This study found 115 (57.5%) uropathogenic *E. coli* strains from patients with UTIs. The frequency of ESBL-positive strains was 52.2%, which was higher than in a similar study on sick patients from three regions in Sudan [11] and Khartoum state [12], where the frequency was 42.0% and 45.2%, respectively. Another study among our population found that *E. coli* is the most frequent gram-negative isolate (54%), and it is resistant to first-line antibiotics [10].

Similar studies reported that the ESBL producer frequency reached 82.5% and 41.1% in patients from India [17,18], 55.5% from Saudi Arabia [19], 66.56% from Nepal [20], 58% from Vietnam [21], 42% from Southwestern Iran [22], 48% from Pakistan [23], and 55.3% from Palestine [24].

On the other hand, ESBL producers were found to be rare in populations from Iran (21%) [9], Malaysia (24%) [25], Kermanshah, Iran (27.3%) [26], Saudi Arabia (23.1%) [27], Iraq (30.7%) [28], and only 6% in Libyans [29].

Because of legal OTC medications, the high frequency of ESBL producers in our population is most likely caused by the incorrect dosage or use of antibiotics for treating UTIs. The majority of patients, especially those in the lower socioeconomic strata, break or stop their treatment regimens due to the drug’s affordability and/or remission of their symptoms during therapy. Doctors typically diagnose UTIs in Sudan primarily based on the relative clinical symptoms present rather than using urine cultures as a regular diagnostic tool unless recurrent infections occur.

The *E. coli* isolates in this study were very resistant to ceftriaxone (81.7%), cefuroxime (81.7%), cefepime (100%), and ceftizoxime (90%) but not as resistant to meropenem (13.3%). Several studies have shown that *E. coli* isolates are resistant to many cephalosporines, such as cefotaxime, ceftriaxone, ceftizoxime, and cefepime [9,19,21,23,25,28]. However, other studies have shown that *E. coli* isolates are sensitive to meropenem [20]. Contrary to our result, all the isolates were found to be resistant to meropenem [2], in addition to other antibiotics such as ceftriaxone, ciprofloxacin, and amoxicillin-clavulanate [3]. In our population, third-generation cephalosporins, like cefotaxime, ceftriaxone, and cefuroxime, didn’t work well for people with *E. coli* that made ESBLs [10-12,30]. In fact, the high prevalence of isolates resistant to third-generation cephalosporins is a serious issue and would reduce the range of available treatments globally. Additionally, these isolates are held accountable for the failure of cephalosporin treatment in UTI patients, several nosocomial outbreaks, numerous deaths, and excessive hospital costs.

Our data showed a high frequency of *bla*_CTX-M_ (85%) and *bla*_SHV_ (70%), compared with *bla*_TEM_ (33.3%). This result was in line with data from Vietnam [21] that showed a high frequency of the CTX-M gene (70%). In another study from Nepal, the CTX-M and TEM genes were found in 86.5% of the isolates [20]. In India, the TEM and CTX-M genes were found to be more common than the SHV gene [18], with 93.47% and 82.6%, respectively, vs. 4.34%. The *bla*_CTX-M_ gene spreads around the world much more than the *bla*_SHV_ and *bla*_TEM_ genes because it can be passed from bacteria in animals (like chickens) to bacteria in people [31].

On the other hand, a previous study found that CTX-M was only found in 6% of isolates and that SHV was not found at all [29]. Other researchers found that CTX-M and TEM were present in only 4% of their isolates [28]. Similar studies in our population [11,12] have found that TEM is common (61%) [5], and CTX-M and SHV are less common (38% and 37%, respectively) [11,12].

There are different results about how often different ESBL producers make class A cephalosporinases. This could be because of the different phenotypes of *E. coli* strains, their patterns of antibiotic resistance on different continents, and the methods used to isolate and describe the ESBL-resistant genes.

A large number of clinically important gram-negative pathogens, such as *E. coli*, have beta-lactamases of class D, which is the main cause of carbapenem resistance [32]. In the current study, our isolates had a reduced (6%) frequency of the class D carbapenemases *bla*_OXA-23_ and *bla*_OXA-51_. This is consistent with findings from Iran [3], where the prevalence of *bla*_OXA-23_ was 10%, and Saudi Arabia [19], where it was 2.7%. A different study found that uropathogenic *E. coli* isolates from Indian patients had low frequencies of the *bla*_OXA-51_ gene and no detection of OXA-23 [33], while ESBL producers in UTI patients from Libya had a high frequency (76.9%) of OXA genes [29].

According to this study’s findings, carbapenem resistance among *E. coli* isolates will be a persistent problem in Sudan. It has been stated that OXA enzymes spread quickly as a result of a number of causative factors, including the diffusion of plasmids, transposons, and integrons across bacterial species, notably gram-negative bacteria. Particularly, the ability of integrons is to bind, spread, and express resistance genes [34].

We were able to identify which *E. coli* strains are resistant to typical UTI treatments using these data. However, the present study has a number of limitations. The sample size from a single tertiary hospital and the number of *E. coli* isolates from urine samples might not be a good representation of the general population or different healthcare settings in terms of the total number of *E. coli* resistance genes and antimicrobial susceptibility and resistance patterns. The study focuses on the *E. coli* resistance gene profile and its distribution across UTIs. It is a laboratory-based study rather than a clinical one. To offer a more thorough understanding of antimicrobial resistance in UPEC isolates in our population, additional research with large sample sizes and diverse health settings is required. Furthermore, the impact of ESBL-producing *E. coli* on treatment outcomes, such as treatment failure rates and patient morbidity and mortality, must be examined in clinically focused studies.

## Conclusions

In this study, the considerable MDR of commonly used cephalosporines and, to a lesser extent, meropenem, the high occurrence of ESBL-producing *E. coli*, and the increased predominance of the *bla*_CTX-M_ and *bla*_SHV_ genes are alarming concerns. In order to effectively treat ESBL producers, it is crucial for the health authorities to manage antibiotic prescriptions based on clinical diagnosis of urine culture, adopt urine culture as the gold standard of UTI identification, demand the implementation of a more sensitive method to detect ESBL-positive isolates for routine susceptibility testing, mandate the clinicians use meropenem as their drug of choice, and implement infection control checklists and bundles in hospitals. There is a need for research focusing on the clinical implications of UTIs caused by isolates of *E. coli* that can produce ESBLs and how resistance can affect treatment, in addition to the underlying mechanisms and risk factors that contribute to the spread of resistance genes in this population. Antimicrobial surveillance studies are also required to develop a prudent antibiotic usage plan and to help direct the clinical management of UTIs.

## References

[1] Zhao Y, Hu K, Zhang J (2019). Outbreak of carbapenem-resistant Acinetobacter baumannii carrying the carbapenemase OXA-23 in ICU of the eastern Heilongjiang Province, China. BMC Infect Dis.

[2] Damavandi MS, Gholipour A, Latif Pour M (2016). Prevalence of Class D carbapenemases among extended-spectrum β-lactamases producing Escherichia coli isolates from educational hospitals in Shahrekord. J Clin Diagn Res.

[3] Pourbaghi E, Doust RH, Rahbar M (2022). Investigation of OXA-23, OXA-24, OXA-40, OXA-51, and OXA-58 genes in carbapenem-resistant Escherichia coli and Klebsiella pneumoniae isolates from patients with urinary tract infections. Jundishapur J Microbiol.

[4] Walker MM, Roberts JA, Rogers BA, Harris PN, Sime FB (2022). Current and emerging treatment options for multidrug resistant Escherichia coli urosepsis: a review. Antibiotics (Basel).

[5] Bastidas-Caldes C, Romero-Alvarez D, Valdez-Vélez V, Morales RD, Montalvo-Hernández A, Gomes-Dias C, Calvopiña M (2022). Extended-spectrum beta-lactamases producing Escherichia coli in South America: a systematic review with a One Health perspective. Infect Drug Resist.

[6] Zhang X, Fang C, Zhang J, Hua W, He R, Zhou M (2022). Carbapenemase- and colistin resistant Escherichia coli strains from children in China: high genetic diversity and first report of blaNDM-5, blaCTX-M-65, blaOXA-10, blaTEM-1, and mcr-1.1 genes co-occurrence in E. coli ST156. Infect Drug Resist.

[7] Elaldi N, Gozel MG, Kolayli F, Engin A, Celik C, Bakici MZ, Vahaboglu H (2013). Community-acquired CTX-M-15-type ESBL-producing Escherichia coli meningitis: a case report and literature review. J Infect Dev Ctries.

[8] Paul D, Ingti B, Bhattacharjee D, Maurya AP, Dhar D, Chakravarty A, Bhattacharjee A (2017). An unusual occurrence of plasmid-mediated bla(OXA-23) carbapenemase in clinical isolates of Escherichia coli from India. Int J Antimicrob Agents.

[9] Farzi S, Ranjbar R, Niakan M, Ahmadi MH (2021). Molecular characterization of antibiotic resistance associated with TEM and CTX-M ESBL in uropathogenic E. coli strains isolated from outpatients. Iran J Pathol.

[10] Saeed A, Hamid SA, Bayoumi M (2017). Elevated antibiotic resistance of Sudanese urinary tract infection bacteria. EXCLI J.

[11] Hisham N Altayb, Mohamed A M Siddig, Nagwa M El Amin (2021). Prevalence of blaCTX-M, blaTEM, and blaSHV Genes among extended-spectrum 𝛽-lactamases-producing clinical Isolates of Enterobacteriaceae in different regions of Sudan. Sudan J Med Sci.

[12] Dirar MH, Bilal NE, Ibrahim ME, Hamid ME (2020). Prevalence of extended-spectrum β-lactamase (ESBL) and molecular detection of blaTEM, blaSHV and blaCTX-M genotypes among Enterobacteriaceae isolates from patients in Khartoum, Sudan. Pan Afr Med J.

[13] Winn WC, Koneman EW, Allen SD (2006). The anaerobic bacteria. Konemans Color Atlas and Textbook of Diagnostic Microbiology.

[14] (2017). CLSI: Performance Standards for Antimicrobial Susceptibility Testing. CLSI supplement M100Published by Wayne, PA: Clinical and Laboratory Standards Institute. https://clsi.org/media/1469/m100s27_sample.pdf.

[15] Queipo-Ortuño MI, De Dios Colmenero J, Macias M, Bravo MJ, Morata P (2008). Preparation of bacterial DNA template by boiling and effect of immunoglobulin G as an inhibitor in real-time PCR for serum samples from patients with brucellosis. Clin Vaccine Immunol.

[16] Hou C, Yang F (2015). Drug-resistant gene of blaOXA-23, blaOXA-24, blaOXA-51 and blaOXA-58 in Acinetobacter baumannii. Int J Clin Exp Med.

[17] Verma S, Kalyan RK, Gupta P, Khan MD, Venkatesh V (2023). Molecular characterization of extended spectrum β-lactamase producing Escherichia coli and Klebsiella pneumoniae isolates and their antibiotic resistance profile in health care-associated urinary tract infections in North India. J Lab Physicians.

[18] Jena J, Sahoo RK, Debata NK, Subudhi E (2017). Prevalence of TEM, SHV, and CTX-M genes of extended-spectrum β-lactamase-producing Escherichia coli strains isolated from urinary tract infections in adults. 3 Biotech.

[19] Wafa A. Alshehri, Tarek A (2021). Extended-spectrum β-lactamase Enterobacteriaceae from patients in Jeddah, Saudi Arabia: antibiotic susceptibility and molecular approaches. J Contemp Med Sci.

[20] Chaudhary MK, Jadhav I, Banjara MR (2023). Molecular detection of plasmid mediated bla(TEM), bla(CTX-M) and bla(SHV) genes in extended spectrum β-lactamase (ESBL) Escherichia coli from clinical samples. Ann Clin Microbiol Antimicrob.

[21] Son TV, Manh ND, Trung NT (2021). Molecular detection of bla(CTX-M) gene to predict phenotypic cephalosporin resistance and clinical outcome of Escherichia coli bloodstream infections in Vietnam. Ann Clin Microbiol Antimicrob.

[22] Ebrahim-Saraie HS, Nezhad NZ, Heidari H, Motamedifar A, Motamedifar M (2018). Detection of antimicrobial susceptibility and integrons among extended-spectrum β-lactamase producing uropathogenic Escherichia coli isolates in southwestern Iran. Oman Med J.

[23] Ehsan B, Haque A, Qasim M, Ali A, Sarwar Y (2023). High prevalence of extensively drug resistant and extended spectrum beta lactamases (ESBLs) producing uropathogenic Escherichia coli isolated from Faisalabad, Pakistan. World J Microbiol Biotechnol.

[24] El Aila NA, Al Laham NA, Ayesh BM (2023). Prevalence of extended spectrum beta lactamase and molecular detection of blaTEM, blaSHV and blaCTX-M genotypes among Gram negative bacilli isolates from pediatric patient population in Gaza strip. BMC Infect Dis.

[25] Fazlul MKK, Farzana Y, Najnin A (2019). Detection of CTX-M-type ESBLs from Escherichia coli clinical isolates from a tertiary hospital, Malaysia. Baghdad Sci J.

[26] Akya A, Ahmadi M, Khodamoradi S (2019). Prevalence of blaCTX-M, blaCTX-M-2, blaCTX-M-8, blaCTX-M-25 and blaCTX-M-3 Genes in Escherichia coli Isolated from urinary tract infection in Kermanshah City, Iran. J Clin Diagn Res.

[27] Mashwal FA, El Safi SH, George SK, Adam AA, Jebakumar AZ (2017). Incidence and molecular characterization of the extended spectrum beta lactamase-producing Escherichia coli isolated from urinary tract infections in Eastern Saudi Arabia. Saudi Med J.

[28] AL-Lami RA, Al-Hayanni HSA, Shehab ZH (2022). Molecular investigation of Some beta-lactamase genes by PCR and DNA sequencing techniques in clinical Escherichia coli. Iraqi J Sci.

[29] Abujnah AA, Zorgani A, Sabri MA, El-Mohammady H, Khalek RA, Ghenghesh KS (2015). Multidrug resistance and extended-spectrum β-lactamases genes among Escherichia coli from patients with urinary tract infections in Northwestern Libya. Libyan J Med.

[30] Dirar M, Bilal N, Ibrahim ME, Hamid M (2020). Resistance patterns and phenotypic detection of β-lactamase enzymes among Enterobacteriaceae Isolates from referral hospitals in Khartoum State, Sudan. Cureus.

[31] Bertrand S, Weill FX, Cloeckaert A (2006). Clonal emergence of extended-spectrum beta-lactamase (CTX-M-2)-producing Salmonella enterica serovar Virchow isolates with reduced susceptibilities to ciprofloxacin among poultry and humans in Belgium and France (2000 to 2003). J Clin Microbiol.

[32] Walther-Rasmussen J, Høiby N (2006). OXA-type carbapenemases. J Antimicrob Chemother.

[33] Chaudhary M, Payasi A (2014). Prevalence, genotyping of Escherichia coli and Pseudomonas aeruginosa clinical isolates for oxacillinase resistance and mapping susceptibility behaviour. J Microb Biochem Technol.

[34] Walsh TR (2010). Emerging carbapenemases: a global perspective. Int J Antimicrob Agents.

